# Supplementary figures and images for: Comparative analysis of silver-nanoparticles and whey-encapsulated particles from olive leaf water extracts: Characteristics and biological activity
Source: PLoS One. 2023 Dec 18;18(12):e0296032. doi: 10.1371/journal.pone.0296032 (PMC10727426; doi:10.1371/journal.pone.0296032)

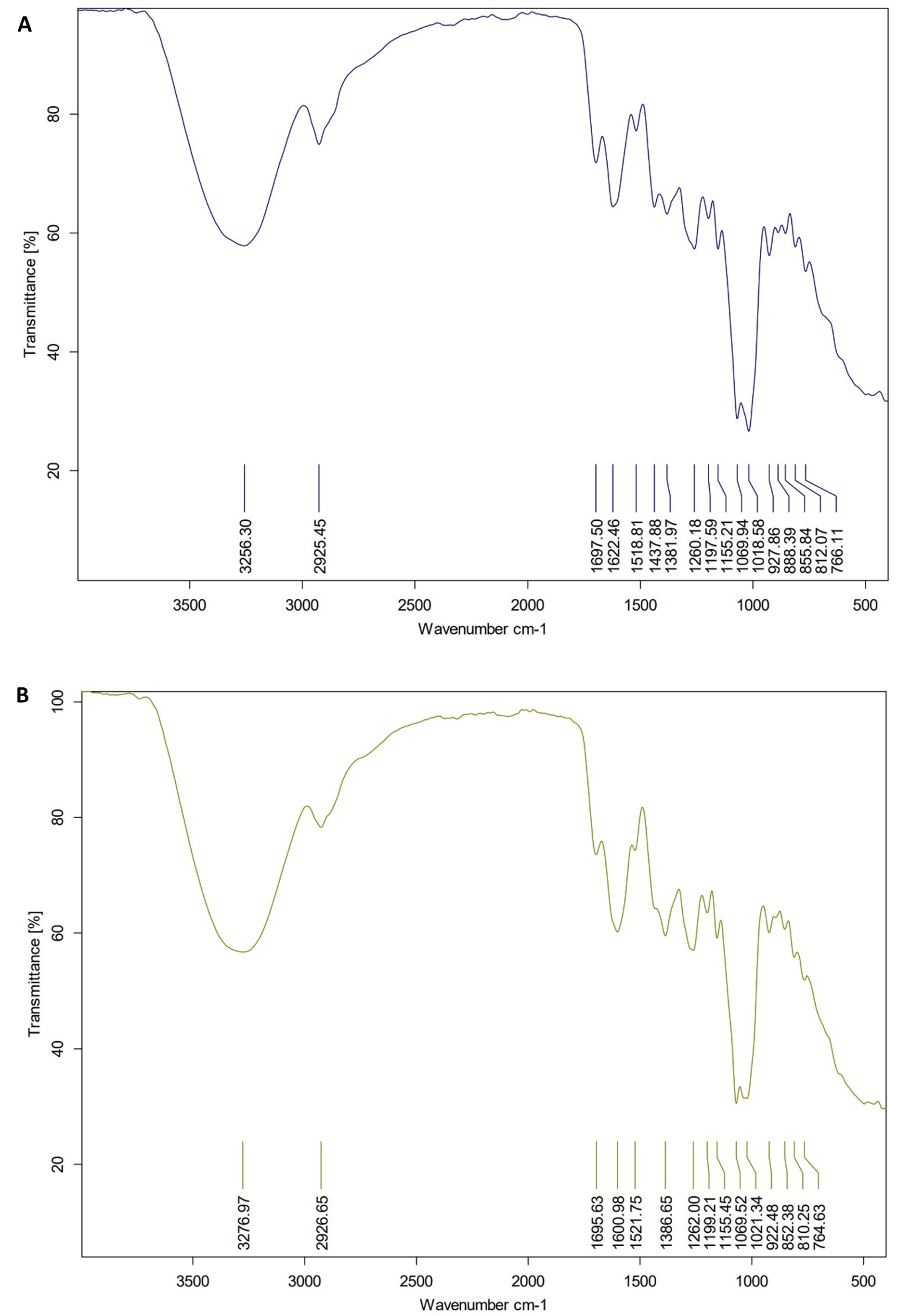

Supplement: S1 Fig — (TIF) [file pone.0296032.s001.tif]

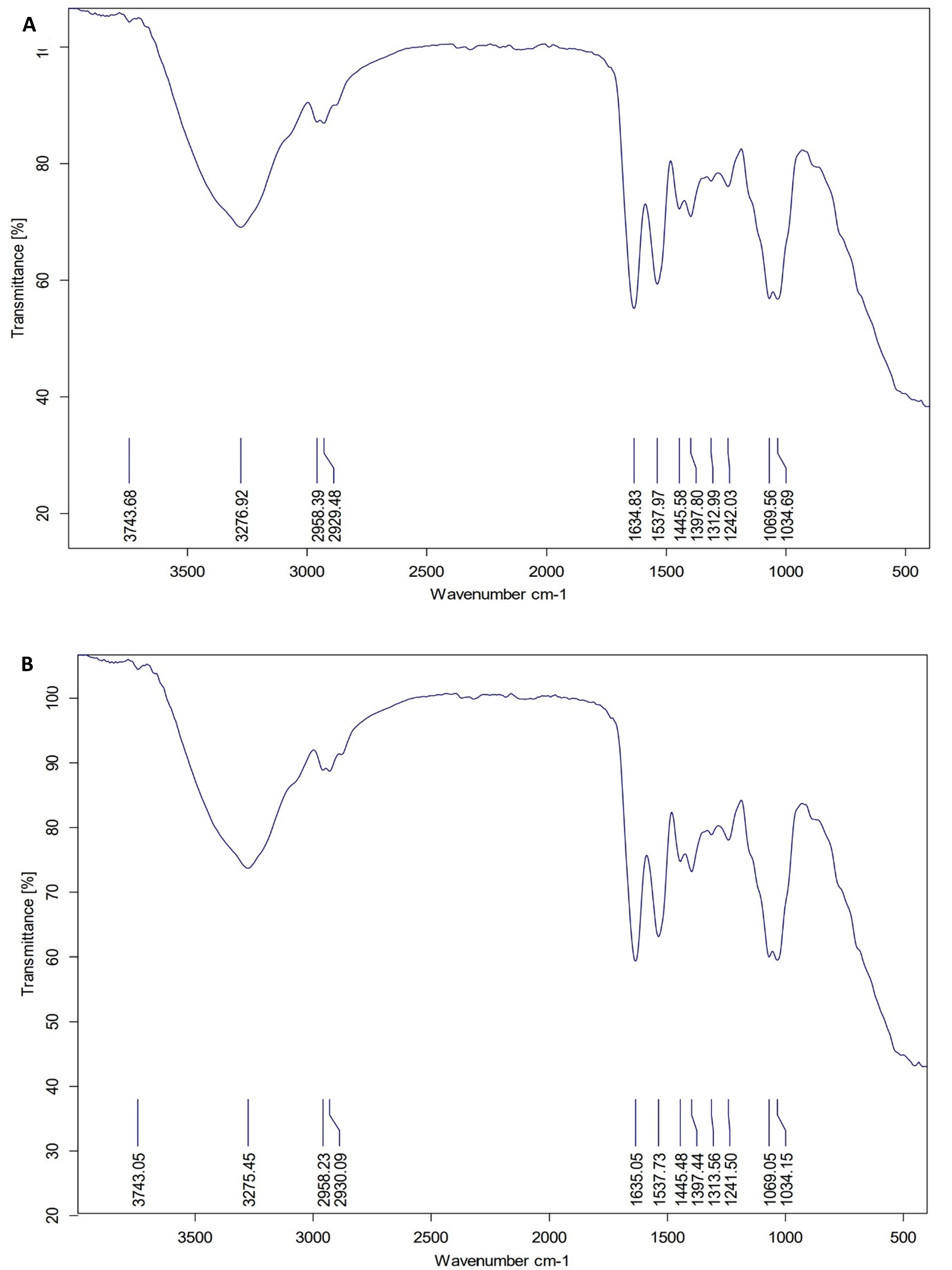

Supplement: S2 Fig — (TIF) [file pone.0296032.s002.tif]

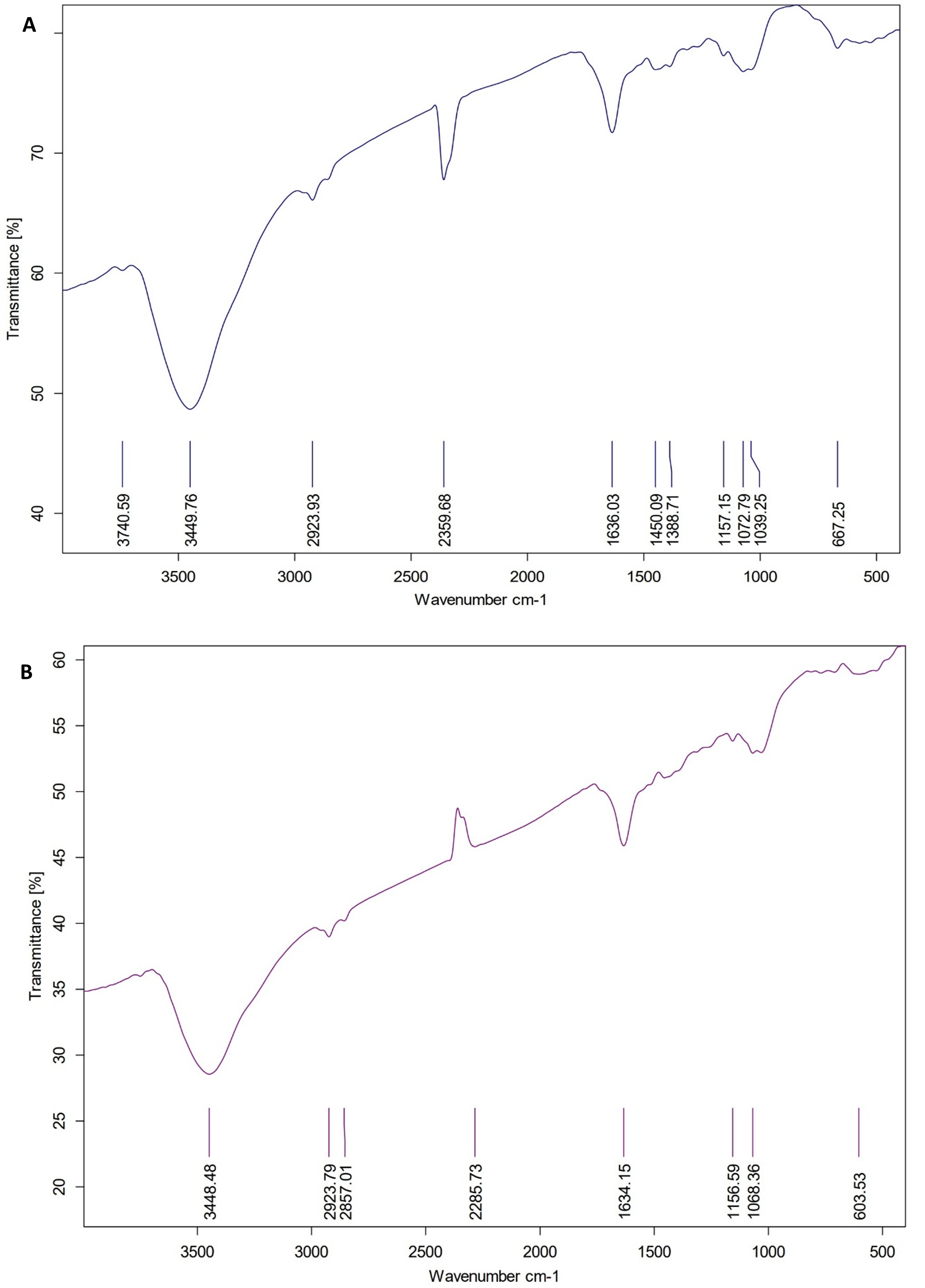

Supplement: S3 Fig — (TIF) [file pone.0296032.s003.tif]
