# Supplementary material for: Comparative analysis of silver-nanoparticles and whey-encapsulated particles from olive leaf water extracts: Characteristics and biological activity
Source: PLoS One. 2023 Dec 18;18(12):e0296032. doi: 10.1371/journal.pone.0296032 (PMC10727426; doi:10.1371/journal.pone.0296032)
Supplement: S1 Table — (DOCX) [file pone.0296032.s004.docx]

| **S1 Table. Bioactive component and antioxidant activity of olive leaf preparations from two cultivars** | | |
| --- | --- | --- |
| **Total phenolics (µg/g)** | | |
| **Preparations/varieties** | **Tofahy** | **Shemlali** |
| **OLE** | 77.81 ± 1.09^aB^ | 95.35 ± 1.73^aA^ |
| **OL/Ag-NPs** | 52.53 ± 2.71^bA^ | 47.78 ± 1.66^bA^ |
| **OL/WPNs** | 12.08 ± 0.22^cB^ | 12.92 ± 0.45^cA^ |
| **Total flavonoids (µg/g)** | | |
| **OLE** | 31.89±0.15^aA^ | 12.66±0.06^aB^ |
| **OL/Ag-NPs** | 18.67±0.42^bA^ | 3.70±0.09^bB^ |
| **OL/WPNs** | 10.79±0.13^cA^ | 3.47±0.03^cB^ |
| **DPPH IC50 (µL/mL)** | | |
| **OLE** | 35.44±0.45^cA^ | 30.39±0.22^cB^ |
| **OL/Ag-NPs** | 55.53±0.77^bB^ | 89.81±0.70^bA^ |
| **OL/WPNs** | 547.52±0.8^aB^ | 769.32±1.11^aA^ |
| **ABTS IC50 (µL/mL)** | | |
| **OLE** | 13.91±0.63^cA^ | 11.99±0.22^cB^ |
| **OL/Ag-NPs** | 37.78±0.57^bA^ | 21.38±1.16^bB^ |
| **OL/WPNs** | 83.76±0.54^aA^ | 71.34±1.05^aB^ |
| **OLE**: Olive leaf extracts; **OL/Ag-NPs:** silver nanoparticles reduced by olive leaf extracts; and **OL/WPNs:** olive leaf extracts encapsulated by whey protein isolate nanoparticles.  The values are means ± SD.  Values with different capital letters (A, B) within the same row indicate a significant difference between olive varieties (*p* < 0.05); Values with different small letters (a-c) within the same column indicate significant differences among different extracts’ preparations (*p* < 0.05). | | |
