# Supplementary material for: Comparative analysis of silver-nanoparticles and whey-encapsulated particles from olive leaf water extracts: Characteristics and biological activity
Source: PLoS One. 2023 Dec 18;18(12):e0296032. doi: 10.1371/journal.pone.0296032 (PMC10727426; doi:10.1371/journal.pone.0296032)
Supplement: S2 Table — (DOCX) [file pone.0296032.s005.docx]

| **S2 Table. Cytotoxicity of olive leaf preparations from two cultivars versus doxorubicin on Vero cells** | | | |
| --- | --- | --- | --- |
| **Tofahy** | | | |
| **Concentration (µg/mL)** | **OLE** | **OL/Ag-NPs** | **OL/WPNs** |
| **0** | 100.00±0.00^aA^ | 100.00±0.00^aA^ | 100.00±0.00^aA^ |
| **31.25** | 99.65±1.13^aA^ | 99.82±1.07^aA^ | 99.73±0.94^aA^ |
| **62.5** | 99.11±1.13^aA^ | 99.29±1.2^aA^ | 99.65±0.98^aA^ |
| **125** | 99.96±1.21^aA^ | 98.58±1.07^aA^ | 47.38±0.53^bB^ |
| **250** | 99.20±0.93^aA^ | 48.89±0.47^bB^ | 11.84±0.35^cC^ |
| **500** | 74.38±1.56^bA^ | 7.89±0.33^cB^ | 6.52±0.8^dB^ |
| **1000** | 10.73±1.36^bA^ | 5.32±0.27^db^ | 4.83±1.07^eb^ |
| **Shemlali** | | | |
| **Concentration (µg/mL)** | **OLE** | **OL/Ag-NPs** | **OL/WPNs** |
| **0** | 100.00±0.00^aA^ | 100.00±0.00^aA^ | 100.00±0.00^aA^ |
| **31.25** | 99.42±1.00^aA^ | 99.65±0.43^abA^ | 99.87±0.93^abA^ |
| **62.5** | 99.29±0.54^aA^ | 98.27±1.09^bA^ | 99.82±0.28^bA^ |
| **125** | 99.07±0.87^aA^ | 42.95±0.83^cA^ | 99.51±0.91^cA^ |
| **250** | 99.69±0.87^aA^ | 20.08±1.63^dB^ | 83.47±1.00^dB^ |
| **500** | 78.77±3.08^bA^ | 7.09±0.60^eB^ | 26.68±2.07^eB^ |
| **1000** | 29.79±1.31^cA^ | 2.75±0.28^fB^ | 2.39±0.13^fB^ |
| **Concentration (µg/mL)** | **Doxorubicin** |  |  |
| **0** | 100.00±0.00^a^ |  |  |
| **3.125** | 99.78±0.13^a^ |  |  |
| **6.25** | 98.18±0.18^a^ |  |  |
| **12.5** | 77.53±0.19^b^ |  |  |
| **25** | 41.05±0.09^c^ |  |  |
| **50** | 28.68±1.61^d^ |  |  |
| **100** | 5.19±0.34^e^ |  |  |
| **OLE**: Olive leaf extracts; **OL/Ag-NPs:** silver nanoparticles reduced by olive leaf extracts; and **OL/WPNs:** olive leaf extracts encapsulated by whey protein isolate nanoparticles.  The values are means ± SD.  Values with different capital letters (A, B) within the same row indicate a significant difference between olive varieties (*p* < 0.05); Values with different small letters (a-c) within the same column indicate significant differences among different extracts’ preparations (*p* < 0.05). | | |  |
