# Supplementary material for: Comparative analysis of silver-nanoparticles and whey-encapsulated particles from olive leaf water extracts: Characteristics and biological activity
Source: PLoS One. 2023 Dec 18;18(12):e0296032. doi: 10.1371/journal.pone.0296032 (PMC10727426; doi:10.1371/journal.pone.0296032)
Supplement: S3 Table — (DOCX) [file pone.0296032.s006.docx]

| **S3 Table. Cytotoxicity of olive leaf preparations from two cultivars versus doxorubicin on HCT-116 cells** | | | |
| --- | --- | --- | --- |
| **Tofahy** | | | |
| **Concentration (µg/mL)** | **OLE** | **OL/Ag-NPs** | **OL/WPNs** |
| **0** | 100.00±0.00^aA^ | 100.00±0.00^aA^ | 100.00±0.00^aA^ |
| **31.25** | 95.46±0.59^bB^ | 99.56±1.11^aA^ | 88.38±1.25^bC^ |
| **62.5** | 79.80±0.96^cB^ | 85.85±0.59^bA^ | 58.81±1.77^cC^ |
| **125** | 26.84±1.93^dA^ | 29.82±0.95^cA^ | 13.71±1.77^dB^ |
| **250** | 10.88±1.18^eA^ | 8.54±0.75^dA^ | 9.32±1.77^eA^ |
| **500** | 8.15±1.42^fA^ | 4.29±0.89^eB^ | 3.03±0.22^fB^ |
| **1000** | 3.07±0.44^gA^ | 3.37±0.39^eA^ | 2.68±0.08^fA^ |
| **Shemlali** | | | |
| **Concentration (µg/mL)** | **OLE** | **OL/Ag-NPs** | **OL/WPNs** |
| **0** | 100.00±0.00^aA^ | 100.00±0.00^aA^ | 100.00±0.00^aA^ |
| **31.25** | 99.90±0.89^aA^ | 99.51±0.59^aA^ | 99.61±0.45^aA^ |
| **62.5** | 99.85±1.02^aA^ | 83.16±0.91^bB^ | 99.41±0.67^aA^ |
| **125** | 78.09±2.49^bB^ | 22.79±1.38^cC^ | 91.26±1.44^bA^ |
| **250** | 35.63±1.28^cB^ | 7.71±1.71^dC^ | 69.25±2.68^cA^ |
| **500** | 13.67±0.97^dA^ | 3.12±0.47^eC^ | 8.98±1.12^dB^ |
| **1000** | 4.83±0.29^eA^ | 3.12±0.22^eB^ | 3.71±0.45^eB^ |
| **Concentration (µg/mL)** | **Doxorubicin** |  |  |
| **0** | 100.00±0.00^a^ |  |  |
| **3.125** | 98.78±0.88^a^ |  |  |
| **6.25** | 81.89±2.13^b^ |  |  |
| **12.5** | 21.77±1.61^c^ |  |  |
| **25** | 13.03±0.44^d^ |  |  |
| **50** | 7.22±1.14^e^ |  |  |
| **100** | 3.37±0.29^f^ |  |  |
| **OLE**: Olive leaf extracts; **OL/Ag-NPs:** silver nanoparticles reduced by olive leaf extracts; and **OL/WPNs:** olive leaf extracts encapsulated by whey protein isolate nanoparticles.  The values are means ± SD.  Values with different capital letters (A, B) within the same row indicate a significant difference between olive varieties (*p* < 0.05); Values with different small letters (a-c) within the same column indicate significant differences among different extracts’ preparations (*p* < 0.05). | | |  |
