# Supplementary material for: Comparative analysis of silver-nanoparticles and whey-encapsulated particles from olive leaf water extracts: Characteristics and biological activity
Source: PLoS One. 2023 Dec 18;18(12):e0296032. doi: 10.1371/journal.pone.0296032 (PMC10727426; doi:10.1371/journal.pone.0296032)
Supplement: S4 Table — (DOCX) [file pone.0296032.s007.docx]

| **S4 Table. Regulatory effect of olive leaf preparations from two cultivars on the expression of TNF-α and Cox1** | | |
| --- | --- | --- |
|  | **TNF-α** | **Cox1** |
| **Preparations** | **Tofahy** | |
| **OLE** | 0.472 ± 0.007^a^ | 2.843 ± 0.103^f^ |
| **OL/Ag-NPs** | 0.398 ± 0.004^b^ | 5.765 ± 0.007^b^ |
| **OL/WPNs** | 0.472 ± 0.001^a^ | 3.613 ± 0.018^e^ |
|  | **Shemlali** | |
| **OLE** | 0.289 ± 0.008^c^ | 7.326±0.006^a^ |
| **OL/Ag-NPs** | 0.305 ± 0.004^c^ | 5.036±0.091^c^ |
| **OL/WPNs** | 0.245 ± 0.001^d^ | 4.536±0.051^d^ |
| **OLE**: Olive leaf extracts; **OL/Ag-NPs:** silver nanoparticles reduced by olive leaf extracts; and **OL/WPNs:** olive leaf extracts encapsulated by whey protein isolate nanoparticles.  The values are means ± SD.  Values with different small letters (a-c) within the same column indicate significant differences among different extracts’ preparations and cultivars (*p* < 0.05). | | |
